# 723 Provincial Burn Mass Casualty Incident Planning: Preliminary Results

**DOI:** 10.1093/jbcr/irad045.198

**Published:** 2023-05-15

**Authors:** Danielle Fuchko, Kathryn King-Shier, Vincent Gabriel

**Affiliations:** University of Calgary, Calgary, Alberta; Calgary Firefighters Burn Treatment Centre, Calgary, Alberta; University of Calgary, Calgary, Alberta; Calgary Firefighters Burn Treatment Centre, Calgary, Alberta; University of Calgary, Calgary, Alberta; Calgary Firefighters Burn Treatment Centre, Calgary, Alberta; University of Calgary, Calgary, Alberta

## Abstract

**Introduction:**

Burn mass casualty incident (BMCI) response planning includes involving relevant stakeholders, assessing regional burn care capacity, and addressing the specialized needs of burn patients. An essential component of these processes is an evaluation of existing resources and plans. In this study, a province with three important elements—a singular health authority to plan for, two burn centres, and updated health technology—was selected for evaluation.

**Aim:**

What are the current policies, protocols, and practices that address a provincial response to a BMCI?

**Methods:**

In this ethics approved case study, qualitative description methodology was used for data analysis. Data were gathered from documents outlining the health system response to a mass casualty incident from the health system internal emergency response plans. Health care professionals directly involved in the response were recruited for interviews. Participants were recruited from prehospital emergency management services, hospital emergency departments, inpatient burn units, and a provincial patient transport department. Interviews were conducted online, recorded, and transcribed. Qualitative description was used to code common themes across documents and transcripts.

**Results:**

Thirteen documents and nine participant interviews were included. Only two documents included burn-specific considerations. Current policies and protocols were limited to all-hazards mass casualty incident planning and did not address specialized needs of burn care. Current plans were based on assumptions that all burn patients would be triaged and transported to burn centres and that surge capacity would be adequate to accommodate for increased demands. Prehospital participants (n=3) felt that existing plans would adequately manage a BMCI. Six participants stated the current plans were not sufficient to manage a BMCI. A comprehensive description of the complex health system response to a potential BMCI was obtained. Some major deficiencies identified included a lack of strategies to bolster burn care staffing capacity such as just-in-time training resources for non-burn trained staff or utilization of outpatient burn clinic staff. Considerations for increased operating room and wound care resources were absent.

**Conclusions:**

Current provincial all hazards response plans are not sufficient to support adequate response to a BMCI and need to be amended to address the specialized needs of burn patients. Strategies for expanding capacity such as care of burn patients at non burn facilities and utilization of outpatient clinics must be integrated into future plans.

**Applicability of Research to Practice:**

The provincial health authority needs to support BMCI response planning efforts to better address this unique hazard prior to an incident occurring for best patient outcomes.

